# Burnout, depression, and suicidal ideation among physicians before and during COVID-19 and the contribution of perfectionism to physicians’ suicidal risk

**DOI:** 10.3389/fpsyt.2023.1211180

**Published:** 2023-07-13

**Authors:** Dafna Kleinhendler-Lustig, Sami Hamdan, Joseph Mendlovic, Yari Gvion

**Affiliations:** ^1^Department of Psychology, Bar-Ilan University, Ramat Gan, Israel; ^2^School of Behavioral Sciences, The Academic College of Tel-Jaffa (MTA), Tel-Jaffa, Israel; ^3^Shaare Zedek Medical Center, Hadassah-Hebrew University School of Medicine, Jerusalem, Israel; ^4^Ministry of Health, Jerusalem, Israel

**Keywords:** physicians, COVID-19, perfectionism, suicidal ideation, suicidal riskfactors, practitioner burnout

## Abstract

**Objectives:**

There is limited data regarding the prevalence of suicidal risk among physicians during COVID-19, and the risk factors relating to it. Dominant risk factors for suicide among physicians are depression and burnout. Maladaptive perfectionism may also serve as a profound risk factor for suicidality among physicians and may aggravate symptoms of distress under the challenges of COVID-19. This study aims to evaluate current suicidal risk, suicidal ideation, depression, and burnout before and during COVID-19 among physicians in Israel, and to identify the best sets of correlates between perfectionism and burnout, depression and suicidal ideation, during these time periods.

**Methods:**

A sample of 246 Israeli physicians (160 before COVID-19 and 86 during COVID-19) completed online surveys assessing lifetime suicidal risk, suicidal ideation during the last year and current suicidal ideation, depression, burnout symptoms and maladaptive perfectionism.

**Results:**

More than one-fifth of the sample (21.9%) reported high suicidal risk (Lifetime suicidal behaviors). More than one-fourth (27.2%) reported suicidal ideation during the last 12 months; and 13.4% reported suicidal ideation during the last 3 months. In addition, more than one-third (34.6%) exhibited moderate–severe levels of depressive symptoms and more than a half of the sample reported burnout symptoms. Maladaptive perfectionism was positively correlated with current suicidal ideation, burnout, and depression. Moderated serial mediation analysis demonstrated indirect effect of perfectionism on suicidal ideation by its impact on burnout and depression only during COVID-19. Before COVID-19, physicians were more likely to experience depressive symptoms.

**Conclusion:**

Physicians in Israel are at increased risk for depression and suicidal ideation, regardless of the COVID-19 pandemic. Maladaptive perfectionism was found to be a risk factor for burnout, depression, and suicidal ideation. During the first waves of the pandemic, physicians were less likely to experience depressive symptoms. However, among physicians who were characterized with high maladaptive perfectionism, depression served as a significant risk factor for suicidal ideation during the pandemic, which places these individuals at increased risk for suicidality. These results highlight the importance of implementing intervention programs among physicians to reduce suicidal risk and to better identify rigid perfectionism and depressive symptoms.

## Introduction

Physicians are routinely exposed to environmental and professional stressors that may increase mental health problems. These include a heavy workload, workplace conflicts, functioning under life and death emergencies, and other stressors associated with depression and suicidal behaviors (1, 2). In addition to these ongoing challenges, the Coronavirus 2019 (COVID-19) outbreak destabilized physicians’ work conditions and sense of security (3, 4) and has led to unprecedented adversities among physicians, who, according to the literature, were an at-risk group for suicidal ideation and behaviors prior to the epidemic (5, 6). Amidst the growing public concern surrounding the impact of the COVID-19 epidemic on physicians’ mental health, it is important to highlight the existing dearth of information regarding the specific effect of COVID-19 on physicians’ suicidal risk (7). Moreover, studies conducted during COVID-19 report high levels of stress reaction symptoms among medical staff, including high frequencies of depression, anxiety (8–11) and burnout (11). However, studies that compare distress symptoms among physicians prior and during COVID-19 are scarce. These findings call for further exploration regarding the prevalence of physicians’ suicidal risk, and the factors relating to it, before and during COVID-19.

### Perfectionism and other risk-factors for suicide among physicians, before and during COVID-19

Although there is abundance of research regarding risk factors for suicide among physicians, there are few studies which examine the effect of personality characteristics, which may be prevalent among physician population, on physicians’ suicidal risk. Specifically, there is a lack of research exploring the interrelationships between multiple risk factors for physician suicidality.

The literature has identified several risk factors that are consistently associated with physician suicidal behaviors. Among the multitude of factors examined in various studies, two have emerged as particularly dominant in their association with physician suicide.

### Depression

Depressive symptoms were reported in high rates among medical trainees and attending physicians compared to the general population (5, 12). These symptoms tend to exacerbate, because physicians’ population usually avoid seeking help for their mental distress (13). Thus, untreated depression was found to be the most dominant risk factor in predicting deaths by suicide among physicians (14).

### Burnout

The World Health Organization defines burnout as an occupational syndrome which is caused by an extreme mental and emotional reaction to work-related stress. Burnout is characterized by high emotional exhaustion, high depersonalization, and a low sense of personal accomplishment (15). Burnout is found in high rates among physicians (16, 17) and has been reported to play a dominant role in predicting depression and suicidal behaviors among this population (18, 19).

### Perfectionism

*Perfectionism* is a personality trait that is considered as a positive quality (20), one that appears to be required to undertake the challenges of physicians’ training. However, research suggests that perfectionism is a multidimensional component which includes maladaptive dimensions that are strongly associated in the general population with depression, hopelessness, and suicidal ideation (21, 22). Frost (1990) defined two of these dimensions as Concern over Mistakes (CM) – the tendency to relate mistakes with personal failure, and Doubts about Action (DA) – tendency to self-doubt and uncertainty. Both were shown to represent aspects of excessive maladaptive perfectionism and were related to symptoms of psychopathology (22). This extreme or maladaptive perfectionism may be prevalent, especially among physicians and medical trainees, owing to their striving for excellence in training, leading them to feel vulnerable and overwhelmed (23). Accordingly, initial evidence demonstrates that maladaptive perfectionism is associated with depression and suicidal ideation among medical students (24). Therefore, expanding the knowledge regarding perfectionism and its contribution to suicidal behaviors is imperative to understand suicide among physicians. During the COVID-19 outbreak, maladaptive perfectionism may have constituted a more salient risk factor for depression and suicide, owing to the uncertainty and the sense of helplessness, which characterized a physicians’ work. The current study explored the effect of maladaptive perfectionism on physician burnout, depression, and suicidal ideation, and sought to identify pathways associated with physician suicidal ideation before and during COVID-19.

### The present study

The study presented in this article was a part of comprehensive research initiated before the COVID-19 outbreak in Israel, which examined risk factors for suicidal risk among physicians. The study evaluates the effect of maladaptive perfectionism on suicidal ideation among physicians, and identifies trajectories from perfectionism to current suicidal ideation, prior and during COVID-19. Also, this article evaluates the frequencies of suicidal ideation and lifetime suicidal risk among physicians in Israel, and examines the prevalence of physicians’ current suicidal ideation, depressive and burnout symptoms before COVID-19 and during the subsequent outbreak. Three hypotheses were tested: (1) Physicians would report higher frequencies of current suicidal ideation, depressive symptoms, and burnout symptoms during COVID-19 than prior to COVID-19. (2) Maladaptive perfectionism would be positively associated with higher frequencies of depressive symptoms, burnout, and current suicidal ideation among physicians. (3) Study findings suggest a predictive model to identify the pathway between maladaptive perfectionism and suicidal ideation. COVID-19 would affect the association between burnout and depressive symptoms with current suicidal ideation among physicians characterized by high maladaptive perfectionism.

## Methods

### Procedures and ethical considerations

Following the receipt of Institutional Review Board (IRB) approval, we commenced the recruitment process by utilizing snowball sampling to gather participants from the pool of physicians. This involved initially approaching Israeli medical organizations and hospitals to access their mailing lists and social media platforms. Snowball sampling allowed us to extend our reach and engage with a wider network of physicians who may be interested in participating in our study. By employing this method, we aimed to ensure a diverse and representative sample of physicians for our research. All respondents participated in the study voluntarily and anonymously, after giving their consent, through computerized self-report questionnaires. All participants were given a referral sheet with the researchers’ contact information, as well as information for mental-health services, if needed.

All translated versions of the measurement tools have been validated in published articles for the Israeli language.

### Materials

1. *Suicidal risk* was measured via the Suicide Behaviors Questionnaire revised (SBQ-R) (25). This instrument comprises four items, each covering a different dimension of suicidality: lifetime suicidal ideation and attempts; the frequency of suicidal ideation over the preceding 12 months; the threat of suicide attempts; self-reported probability of suicidal behavior in the future. Items scored on a Likert scale were summed for a final score, that ranged from 3 to 18. High scores represent greater risk for suicidal behaviors. A score of 7 or higher is considered as screening positive for “*lifetime suicidal risk*,” because it maximizes the rates of sensitivity and specificity for lifetime suicidal risk in non-clinical samples (25). In this study, the internal consistency of the SBQ-R was acceptable (*α* = 0.73).
  To assess suicidal ideation during the last year, the second item in the SBQ-R “*How often have you thought about killing yourself in the past year?”* was used. The responses range from 1 (“never”) to 5 (“very often – five times or more”). In the current study, any response over 1 (at least once) was considered positive for suicidal ideation during the last 12 months.
2. *Current suicidal ideations* were estimated by the question: “*During the last three months have you experienced thoughts about killing yourself / end your life? (Check: Yes/No).”* Current suicidal ideation was calculated for any participants who checked “Yes” for experiencing suicidal ideation during the last 3 months.
3. *Depression* was assessed with the standardized and validated PHQ-8 (26). The total score is between 0 and 24 points. A total score of 0–4 represents no significant depressive symptoms. A total score of 5–9 represents mild depressive symptoms; 10–14, moderate; 15–19, moderately severe; and 20–24, current severe depression (27). In the study, a cut-off score of 10 or higher was used in order to screen positive for depressive symptoms, since it was useful in maximizing the sensitivity and specificity rates for measuring moderate–severe symptoms of depression (28). In this study, the internal consistency of PHQ-8 was good (*α* = 0.84).
4. *Burnout* symptoms were assessed via Maslach Burnout Inventory-Human Services Survey (MBI-HSS) (29). This 22-item questionnaire has three subscales evaluating three domains of burnout: emotional exhaustion (EE)- nine items (e.g., “*I feel emotionally drained from my work”*), depersonalization (DP) – five items (e.g., “*I do not really care what happens to some patients”*), and personal accomplishments (PA) – eight items (e.g., “*I feel I’m positively influencing other people’s lives through my work”*). All items are scored from 0 to 6 (“never” to “every day”). In this study, the added sum of EE and DP scales were used to measure burnout. The PA scale was not used in data analysis since it was considered in previous research to be less associated as a measure of burnout symptoms that are caused by work-related stress (30). In this study the cut-off scores of each scale, representing various aspects of burnout symptoms, were defined by norms for medical professionals used in previous research. Thus, screening positive for EE is defined as total score of 27 and above at this subscale, and screening positive for DP is defined as total score of 10 and above at this subscale (29). In this study, the internal consistency of MBI total score was excellent (*α* = 0.90), and the internal consistencies of each of the scales were good (EE *α* = 0.79, DP *α* = 0.88).
5. *Maladaptive perfectionism* was measured with The Frost Multidimensional Perfectionism Scale (FMPS), which is one of the most validated and widely used questionnaires for measuring perfectionism (31). It includes 35 items rated on a 5-point Likert-type scale, and measures six dimensions of perfectionism, which considered adaptive and maladaptive. In the current study the added sum of two maladaptive perfectionistic attitudinal scales were used: Concern over mistakes (CM) and Doubts about action (DA). In this study, the internal consistency of the two scales’ total score was good (*α* = 0.87), and the internal consistencies of each of the scales were acceptable (CM *α* = 0.87, DA *α* = 0.76).
6. *Demographic data*, including stage of professional training (internship, residency, or attending physicians), gender, age, and marital status were obtained from all participants. The study’s time of participation was divided into two groups: before COVID-19 (participation before March 2020—the beginning of the first quarantine in Israel) and during COVID-19 (participation after the beginning of the first quarantine).

### Data analysis

Data were analyzed using IBM SPSS statistics version 26 (32), with an alpha level of 0.05 for all statistical tests. First, data were analyzed for missing data. Little’s Missing Completely at Random test (4MCAR test) (33) was non-significant, χ^2^ (19) = 19.76, *p* = 0.409, indicating that data were missing completely at random. Therefore, the maximum likelihood was used to manage the missing data, which was lower than 1.5% for all values. Differences of background variables by time (before and during COVID-19) were analyzed. An independent sample t-test was used for continuous variables and a Chi-square (χ^2^) test for independence or Fisher’s exact test [FIT] for categorical variables were used. In addition, Zero-order correlations were performed for associations among continuous variables. Lastly, moderated serial mediation analysis was conducted using the PROCESS macro for SPSS [model 92; (34)] in order to examine the best fitting moderated-mediation model for suicidal ideation during the last 3 months (No versus Yes). Percentile confidence intervals (CI) were estimated for the indirect effects based on 5,000 bootstrap samples of the study’s group, beyond time (before and during COVID-19). In the analysis, maladaptive perfectionism served as the independent variable, burnout and depression scores as the mediators (in serial), time (dummy coded: 0 = before COVID-19; 1 = During COVID-19) as the moderator and SI during the last 3 months as the dependent variable. Family status, stage of training (two dummy coded variables: resident and attending with intern as the reference group for both dummy variables), age and gender, served as covariates. Following Aiken and West (1991), maladaptive perfectionism, burnout and depression were mean-centered.

## Results

### Sample

The sample included 246 physicians from various internships and hospitals in Israel who completed the survey (11.4% interns, 52.8% residents, and 35.8% attendings). Slightly above one-third (35%) of participants completed the survey after the initial COVID-19 outbreak in Israel on March 2020 (between the end of April 2020 and January 2021), while 65% of the participants completed the survey prior to the outbreak (between January and the beginning of March 2020). Most participants were married (82.5%), and their mean age was 37.75 (SD = 9.10). There were no significant differences in demographic data among the two groups (before and during COVID-19) (*p* > 0.05) (Table 1).

**Table 1:** Background and clinical characteristics of the study groups.

	Time							
	Total sample		Before COVID-19		During COVID-19			
Characteristic	(*N* = 246)		(*n* = 160)		(*n* = 86)			
*Background*	*M*	*SD*	*M*	*SD*	*M*	*SD*	*t*	*P*
Age	37.75	9.10	36.96	8.23	39.21	10.42	−1.73	0.086
	**n**	**%**	**n**	**%**	**N**	**%**	**χ** ^2^	**P**
Gender								
Male	92	37.4	56	35.0	36	41.9	1.12	0.289
Female	154	62.6	104	65.0	50	58.1		
Family status								
Not married	43	17.5	29	18.1	14	16.3	0.13	0.716
Married	203	82.5	131	81.9	72	83.7		
Stage of training								
Intern	28	11.4	16	10.0	12	14.0	0.97	0.615
Resident	130	52.8	87	54.4	43	50.0		
Attending	88	35.8	57	35.6	31	36.0		
**Clinical**	**M**	**SD**	**M**	**SD**	**M**	**SD**	**t**	**P**
Maladaptive perfectionism: Concern over mistakes	26.72	6.70	27.14	6.77	25.93	6.54	1.35	0.177
Doubts about action	10.81	3.52	11.05	3.43	10.36	3.67	1.47	0.144
Burnout	**38.04**	**16.25**						
Depression	**7.0**	**5.31**						
Suicidal ideation for the last year	**1.45**	**0.95**						
	**n**	**%**	**n**	**%**	**N**	**%**	**χ** ^2^	**P**
Lifetime suicidal risk^a^	54	22.0	40	25.0	14	16.3	2.48	0.115
Suicidal ideation during the last year^b^	67	27.2	45	28.1	22	25.6	0.18	0.669
Suicidal ideation during the last 3 months^c^	33	13.4	24	15.0	9	10.5	0.99	0.320
Moderate/severe depression^d^	85	34.6	63	39.4	22	25.6	4.71	0.030
Burnout: Emotional exhaustion	139	56.5	94	58.8	45	52.3	0.94	0.332
Depersonalization	125	50.8	81	50.6	44	51.2	0.01	0.936

^a^Values represent total score of High Suicidal Risk (SBQ-R) > =7. ^b^Values represent the positive category (at least one suicidal ideation). ^c^Values represent total score of Moderate/Severe Depression (PHQ-8) > =10. ^d^Values represent total score of Emotional exhaustion (EE) > =27 and Depersonalization (DP) > =10 in the MBI. M, Mean; SD, Standard Deviation. Values present the mean and standard deviation of the total scores of: MBI, PHQ (8), and SBQ-R second item accordingly.

### Participant clinical characteristics

Of all participants, 22.0% screened positive for lifetime suicidal risk, 27.2% had experienced suicidal ideation at least once during the previous year, and 13.4% had experienced suicidal ideation during the last 3 months. Moreover, 34.6% of all participants reported moderate to severe depressive symptoms, 56.5% had screened positive for emotional exhaustion, and 50.8% for depersonalization symptoms. Physicians were more likely to experience depressive symptoms before the outbreak than during COVID-19. No other significant differences were found before and during COVID-19 (all *p*’s > 0.05) (Table 1).

### Correlations between study variables

Current suicidal ideation was positively correlated with maladaptive perfectionism, burnout, and depression before and during COVID-19. Moreover, burnout and depression were positively associated with maladaptive perfectionism and each other, before and during COVID-19. Correlations were found among perfectionism, burnout, depression, and suicidal ideation (during the last 12 and 3 months) before and during COVID-19 (Table 2).

**Table 2:** Means, standard deviations, and correlations among study variables by time.

Variable		1	2	3	4	5	*M*	*SD*
1	Maladaptive perfectionism	–	0.41^***^	0.49^***^	0.32^***^	0.21^**^	38.19	8.53
2	Burnout	**0.57** ^***^	–	0.53^***^	0.34^***^	0.14	39.14	15.71
3	Depression	**0.57** ^***^	**0.58** ^***^	–	0.30^***^	0.19^*^	8.45	4.93
4	Suicidal ideation for the last year	**0.35** ^***^	**0.29** ^**^	**0.61** ^***^	–	0.53^**^	1.46	0.83
5	Suicidal ideation for the last three months	**0.28** ^**^	**0.37** ^***^	**0.53** ^***^	**0.64** ^***^	–		

Pearson coefficients are presented for correlations between continuous variables. For correlations between dichotomous and continuous variables, point-biserial coefficients are presented. Correlations for before COVID-19 (*n* = 160) are shown above the diagonal, and correlations for during COVID-19 participants (*n* = 86; emphasized with bold letters) are shown below the diagonal. Means (M) and Standard Deviations (SD) for before COVID-19 are shown in the vertical columns and Means and Standard Deviations for during COVID-19 are shown in the horizontal rows. Means and standard deviations are presented only for continuous variables. ^*^p < 0.05; ^**^p < 0.01; ^***^p < 0.001.

### Moderated serial mediation analysis model

**Figure 1:**
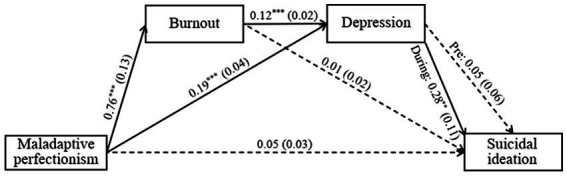
Moderated serial mediation model depicting the indirect effect of maladaptive perfectionism on suicidal ideations conditional to time (before and during COVID-19). *N* = 246. Values are unstandardised regression. In parentheses: standard errors. Solid lines indicate significant paths and dashed lines indicate nonsignificant paths. Pre, Before COVID-19; During, During COVID-19. ^**^p < = 0.01. ^***^p < = 0.001.

As presented in the model (Figure 1), the interaction between depressive symptoms and time on current suicidal ideation was marginally significant (*B* = 0.24, *SE* = 0.12, *p* = 0.051), showing a positive and significant association during COVID-19 compared to non-significance before COVID-19 time period.

The effect of maladaptive perfectionism on burnout and depression was positive and significant, and not statistically different between before and during COVID-19 (*B* = 0.24, *SE* = 0.21, *p* = 0.248 and *B* = 0.02, *SE* = 0.07, *p* = 0.828, respectively). The effect of maladaptive perfectionism on current suicidal ideation was non-significant and was not moderated by time (*B* = −0.10, *SE* = 0.07, *p* = 0.168).

In addition, the effect of burnout on depression was positive and significant, and not statistically different between before and during COVID-19 (*B* = 0.002, *SE* = 0.04, *p* = 0.966). The effect of burnout on suicidal ideation was non-significant and was not moderated by time (*B* = 0.04, *SE* = 0.04, *p* = 0.390).

As for the conditional indirect effects, the indirect effect of maladaptive perfectionism on suicidal ideation via burnout and depression was non-significant before COVID-19 (*B* = 0.004, *SE* = 0.01, bootstrapped 95% CI: −0.01, 0.02). As mentioned above, percentile confidence intervals (CI) were estimated for the indirect effects based on 5,000 bootstrap samples of the study’s group. In contrast, the serial indirect effect was positive and significant during COVID-19 (*B* = 0.04, *SE* = 0.17, bootstrapped 95% CI: 0.01, 0.58), meaning, that higher maladaptive perfectionism predicted higher burnout, which in turn predicted higher depression, which subsequently predicted higher likelihood of current suicidal ideation. Moreover, the index of moderated mediation was significant (*B* = 0.03, *SE* = 0.17, bootstrapped 95% CI: 0.003, 0.57), thus supporting the moderating role of time on the serial indirect effect. Controlling for demographic characteristics of age, gender, family status, and stage of training did not contribute to any statistically significant changes in the model, with one exception of the direct effect of maladaptive perfectionism on current suicidal ideation, which became marginally significant (*B* = 0.06, *SE* = 0.03, *p* = 0.080).

## Discussion

This study forms part of a broader cross-sectional research endeavor aimed at investigating the relationships between personal and professional risk factors and their impact on physicians’ suicidal thoughts and behaviors. During the initial phase of participant recruitment, COVID-19 emerged in Israel, presenting physicians with unprecedented and challenging circumstances that had a profound effect on both them and their environment. It is reasonable to assume that individual characteristics would influence the stress response and distress experienced by physicians in the face of these adversities. Consequently, this article presents a unique opportunity to explore the association between maladaptive perfectionism and burnout, depression, and suicidal ideation among physicians, both before and during the COVID-19 pandemic. Additionally, this study seeks to estimate the prevalence of suicidal risk and suicidal ideation among physicians during these distinct time periods, offering valuable insights into the impact of the pandemic on their mental well-being.

### Maladaptive perfectionism and its relation to burnout, depression and suicidal ideation

A main aim of the current study was to explore the effect of maladaptive perfectionism on physicians’ distress and suicidality. Our hypothesis posited that perfectionism would be a prominent risk-factor under the uncertainty and frustration that took place in physicians’ working environment during the initial outburst of COVID-19 (35). Hence, a model was suggested that would examine how maladaptive perfectionism effects physicians’ current suicidal ideation through its effect on burnout and depressive symptoms before and during COVID-19. Indeed, high maladaptive perfectionism is associated with higher rates of burnout, which in turn is associated with higher rates of depressive symptoms, before and during COVID-19. These results complement the literature discussing the risk that rigid and extreme forms of perfectionism poses for physicians’ mental health (23). While the correlation between maladaptive perfectionism and suicidal ideation was found to be highly significant, it is important to note that maladaptive perfectionism did not show a direct association with suicidal ideation, both before and during the COVID-19 period. This suggests that maladaptive perfectionism alone does not independently contribute to the prevalence of suicidal ideation. However, an intriguing finding emerged during the COVID-19 period, where burnout and depression mediated the relationship between maladaptive perfectionism and suicidal ideation. This indicates that, specifically during this challenging and stressful time, high levels of maladaptive perfectionism may lead to suicidal ideation through the pathway of increased depression. In essence, the interplay between maladaptive perfectionism, burnout, and depression becomes particularly salient during times of heightened stress, such as the COVID-19 pandemic. Considering the overall reduction in symptoms of depression among the sample during the initial waves of COVID-19, it was concluded that during these times it is imperative to identify physicians who are characterized with high maladaptive perfectionism, because they could be at an increased risk for depression and suicide. Interventions aimed at treating physicians who suffer from depression should consider perfectionism a central factor for change that will lessen suicidal risk.

### Suicidal risk among physicians before and during COVID-19

Although research evidence and literature reviews over the years suggest that physicians are vulnerable to mental disorders and suicidal thoughts and behaviors, there is a lack of updated evidence-based data regarding physicians’ suicidal risk, especially during the COVID-19 outbreak (36). Moreover, recent publications that studied physician population prior to COVID-19 have reported contradictory findings regarding physician suicide rates, such as a decline in suicide over the last decades compared to the general population (26, 37, 38). Thus, there is a need to further investigate physician suicidal behaviors rates, as well as the prevalence rates of central risk-factors that contribute to suicidality among physicians. The current study’s results demonstrate high prevalence of lifetime suicidal risk among physicians in Israel, compared to other findings among the general population (39). These results are compatible with some evidence worldwide regarding physicians’ suicidal risk (40). The findings suggest prevalence rates of prolonged and current suicidal ideation among medical professionals in Israel, which are higher than other reported suicidal ideation rates among the general population (41–43) and among physicians worldwide, before as well as during the initial outbreak of COVID-19 (44). The high rates of suicidal ideation found in the current study among physicians highlight the importance of further investigation of the specific working conditions in the country’s medical system. A possible explanation for the elevation in suicidal ideation among the study’s population, could be related to several incidents of physician deaths by suicide that had occurred in Israel close in time during the year preceding the study, which had a shocking effect on physicians. Both the research and clinical literature suggest an emotional “contagion” in suicide (45, 46), which approximates the time-period of the current research that may have affected the results (47).

Contrary to the initial hypothesis of the reported study, there were no significant differences in suicidal ideation among physicians before and during the outbreak. There is a need to further explore possible explanations for these findings, perhaps through narrative studies and interviews with physicians. One explanation may be related to the fact that the study was conducted during the first waves of the COVID-19 outbreak in Israel, and that physicians may have felt a sense of meaning and importance in their work. These results resonate with Joiner’s “pulling together” phenomena that occurs during initial outbreaks and other times of crisis (48). Other explanations could be related to individual characteristics and predispositions that were not explored in this study, such as physicians’ years of professional experience (49), and history of military service, which is mandatory in Israel and may provide more resiliency in times of crisis (50). The results are compatible with other studies that found no changes concerning suicidality before and during the first waves of COVID-19 among the general population (51, 52). Some studies even found a reduction in suicidal behaviors during the initial outburst of the pandemic (53). However, studies that have been conducted during later stages of the epidemic among the general population reported an initial reduction of suicidality, followed by a rise of suicide cases during later waves (54, 55). Accordingly, medical staffs may have experienced a lapse in the sense of meaning and importance in later waves of the pandemic (56), while still facing its enduring challenges (57). That change may aggravate their distress and burnout, and eventually expose them to additional risk for suicide.

### Depressive symptoms and burnout before and during COVID-19

High rates of depressive symptoms were found among the study participants, regardless of COVID-19. These rates are higher than those reported among the general population (58), which included the outbreak in Israel and worldwide (59). However, they are compatible with some recent studies among physicians and medical trainees (60, 61). About one-half of the total sample reported experiencing clinically significant burnout symptoms, that are associated with depression and suicidal ideation (62). The findings call for further investigation regarding the professional and individual reasons for which many physicians experience substantial mental distress.

The present findings suggest that physicians’ distress during the first months of the COVID-19 outbreak did not change significantly and, to some extent, might have improved, as shown by the reduction in rates of depressive symptoms among the sample. Although evidence suggest high rates of burnout, anxiety, and depression among physicians during the outbreak (11), these high frequencies of depression and other mental disorders may reflect physicians’ overall mental health beyond the impact of COVID-19. Few studies have reported that during the first waves of the pandemic, physicians who were treating COVID-19 patients experienced lower levels of burnout symptoms (63, 64). It can be postulated that physicians who worked during the initial waves of COVID-19 held a higher sense of purpose, as has been shown in studies regarding other outbursts (56). Moreover, during the beginning of the COVID-19 crisis, physicians in Israel were given a wider systematic and social support, as the public and the media applauded their work, aiding medical staffs to improve their working conditions. In addition, during COVID-19 initial outbreak, many hospitals made systematic changes in physicians’ schedules and shifts (65), which may have minimized sleep deprivation. Moreover, hospitals provided medical staffs with personal protective equipment that could have reduced anxiety symptoms (66). These changes may have had a positive impact on physicians’ mental state and well-being.

## Conclusion

In the current study, high rates of suicidal ideation, depression and burnout symptoms were found among physicians in Israel, before and during COVID-19. These results reflect the vulnerability of physicians to mental distress, and the need to engage in systematic interventions to promote their overall well-being, beyond the specific challenges of COVID-19. These results highlight the importance of recognizing maladaptive perfectionism as a risk factor for poor mental health outcomes in physicians, especially during times of heightened stress and adversity. The identification and implementation of effective intervention programs should take into account the impact of maladaptive perfectionism and its potential role in exacerbating mental health challenges among physicians. By addressing maladaptive perfectionism within such programs, healthcare organizations and institutions can better support physicians’ well-being and mitigate the risk of suicidal ideation. Despite the adversities of the initial outbreak, physicians’ overall mental state during this time period had not changed and even improved in certain aspects. It can be assumed that the social support provided for physicians during the initial waves of the pandemic, had a central role in protecting their well-being. However, considering the provision of routine services needed during the prolonged battle against COVID-19, the public and systematic support physicians are receiving is thinning, while their workload continues to rise. This could place them in additional risk for mental illness and suicidality.

### Limitations

The study has several limitations: First, is the use of self-reported measures. The self-reporting in the questionnaires might have affected the findings of high depression/suicidal ideation. We suggest that future research concerning physicians’ suicidal ideation should include standardized interviews to make psychiatric diagnoses. Second is the study’s cross-sectional design, which limits conclusions regarding causality. Longitudinal studies could overcome this prominent issue in future studies. Moreover, as the survey was distributed through e-mail and social media to hundreds of physicians, it was challenging to determine the study’s exact response rate. Still, it can be assumed it was substantially low, in accordance with other studies concerning physicians (67). While the recruitment method employed in our study is commonly accepted in research conducted among the physician population, it is important to acknowledge the limitations associated with the relatively small sample size and snowball sampling technique. These limitations restrict the generalizability of the findings and may introduce sampling bias, potentially impacting the accurate representation of symptoms of psychopathology among physicians. Additionally, it is worth noting that the questionnaire was distributed a few months prior to the onset of the COVID-19 outbreak in Israel, and during the initial waves of contagion. As the pandemic unfolded, there were significant changes in the work environment of physicians and the overall public response to the crisis, which could have influenced the results. Therefore, the findings of our study reflect the initial reaction of the physician population to the outbreak. Future research may provide a more comprehensive understanding of the mental state of physicians across different stages of the pandemic, allowing for a more nuanced assessment of the long-term impact of COVID-19 on physicians’ mental well-being. Finally, the relatively small sample size of physicians collected during the pandemic prevents further analysis of differences between subgroups of interest, such as first-year interns or intensive care unit physicians who were treating most cases of COVID-19 patients. However, findings from other studies in Israel that were conducted during the pandemic, suggest that emergency department physicians reported lower levels of burnout compared to physicians in internal departments, even though they were more exposed to COVID-19 patients (50).

Nonetheless, to the best of our knowledge, the current study is one of the first to address the contribution of maladaptive perfectionism to physicians’ mental distress and suicidal risk. Also, it is one of the first to provide updated information regarding suicidal risk among physicians in Israel, and to estimate the effects of COVID-19 on physicians’ suicidal ideation and depression, compared to their mental state prior the pandemic. The results demonstrate that the battle against COVID-19 was a part of a broader picture of physicians’ adversities and mental distress. Hence, there is a need for organization-directed interventions that will promote physicians’ supportive working environments during their routine work, as well as during crisis. The results also highlight the role of maladaptive perfectionism in the development and maintenance of burnout, depressive symptoms, and suicidal ideation among physicians. These findings are applicable to better identify physicians at risk and to promote intervention programs that will moderate rigid perfectionism.

## Data availability statement

The raw data supporting the conclusions of this article will be made available by the authors, without undue reservation.

## Ethics statement

The studies involving human participants were reviewed and approved by the Institutional Review Board (IRB) of Bar-Ilan University. The patients/participants provided their written informed consent to participate in this study.

## Author contributions

DK-L and YG were responsible together with SH and JM for the ideas, formulation of overarching research goals and aims, design of methodology, and supervision of DK-L. DK-L conducted the research, analyzed the data, and wrote the first draft. All authors contributed to the article and approved the submitted version.
